# Dynamic Navigation in Dental Implantology: The Influence of Surgical Experience on Implant Placement Accuracy and Operating Time. An in Vitro Study

**DOI:** 10.3390/ijerph17062153

**Published:** 2020-03-24

**Authors:** Gerardo Pellegrino, Pierantonio Bellini, Pier Francesco Cavallini, Agnese Ferri, Andrea Zacchino, Valerio Taraschi, Claudio Marchetti, Ugo Consolo

**Affiliations:** 1 Oral and Maxillofacial Surgery Division, DIBINEM, University of Bologna, 40125 Bologna, Italy; gerardo.p@libero.it (G.P.); agnese.ferri@ymail.com (A.F.); andrea.zacco@hotmail.it (A.Z.); v.taraschi@bresmedical.com (V.T.); claudio.marchetti@unibo.it (C.M.); 2 Division of Oral and Maxillofacial Surgery, Dental Clinic, University of Modena and Reggio Emilia, 41125 Modena, Italy; pierantonio.bellini@unimore.it (P.B.); pfcavallini@yahoo.it (P.F.C.)

**Keywords:** dynamic navigation, image-guided surgery, dental implants, computer-guided implantology

## Abstract

Aim: the aim of this in vitro study was to test whether the implant placement accuracy and the operating time can be influenced by the operator’s experience. Materials and methods: sixteen models underwent a (Cone Beam Computer Tomography) CBCT and implant positioning was digitally planned on this. The models were randomly assigned to four operators with different levels of surgical experience. One hundred and twelve implant sites were drilled using a dynamic navigation system and operating times were measured. Based on postoperative CBCTs, dental implants were virtually inserted and superimposed over the planned ones. Two-dimensional and 3D deviations between planned and virtually inserted implants were measured at the entry point and at the apical point. Angular and vertical errors were also calculated. Results: considering coronal and apical 3D deviations, no statistically significant differences were found between the four operators (*p* = 0.27; *p* = 0.06). Some vectorial components of the deviation at the apical point and the angular errors of some operators differed from each other. Conclusions: within the limitations of this study, dynamic navigation can be considered a reliable technique both for experienced and novice clinicians.

## 1. Introduction

Computer-guided surgery is a technique that allows for the positioning of dental implants based on a virtual preoperative plan. With respect to conventional free-hand implant placement, computer-guided implantology brings many advantages, like prosthetic-driven implant placement, the simplification of some surgical procedures, keeping them minimally invasive, the reduction in operative times and, mainly, a more accurate implant placement [1,2,3,4]. Computer-guided surgery can be divided into two techniques: the static one, using surgical templates, and dynamic navigation. Dynamic navigation makes use of systems working with a camera recording the position of the patient and the surgical instruments, and a screen displaying the position of the drills onto Cone Beam Computer Tomography (CBCT) images in real-time during surgery [5]. Both techniques have good accuracy values reported in the literature [3,6,7], but the majority of the studies involved skilled operators or failed in reporting their level of experience. Some model-based studies investigated whether surgical experience could influence the accuracy of implant placement using drill guides [8,9]. The literature is not consistent, although similar values of errors between inexperienced and skilled surgeons were demonstrated, and a great improvement in accuracy was shown in novice groups when using the guided method [9,10]. The accuracy reported in studies involving dynamic systems is similar to that gained with a static technique, but few studies on dynamic navigation focused on the experience factor [11,12]. Moreover, no studies investigated the difference in implant placement accuracy between expert surgeons in dynamic navigation and operators who were trying it for the first time. Therefore, the aim of this study was to test the equivalence of implant placement accuracy values using dynamic navigation between operators with different levels of experience and knowledge of this guided technique. The secondary aim was to evaluate the difference in drilling time.

## 2. Materials and Methods

**Figure 1:**
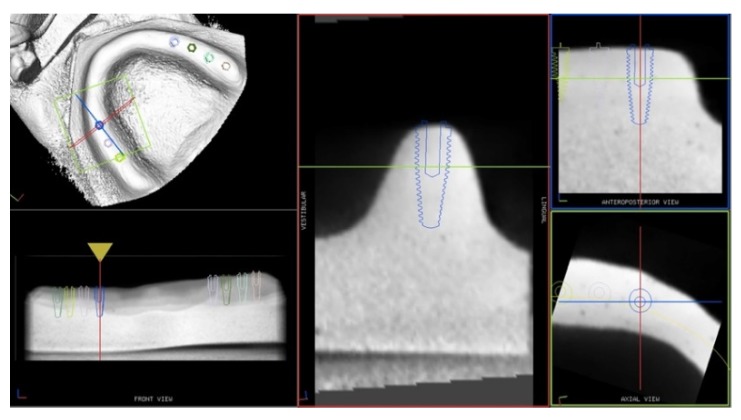
Plan of the implants position on the model CBCT images.

A randomized in vitro study was designed following the CRIS (Checklist for Reporting In-vitro Studies) statement guidelines [13]. Sixteen extra-hard plaster models were made, reproducing the same edentulous maxilla. CBCT scans were done with a reference tool positioned on them and the DICOM files were imported to the navigation system software. For each model, seven implants were virtually planned on the CBCT images to reach the needed sample size of 112 (Figure 1). 

**Figure 2:**
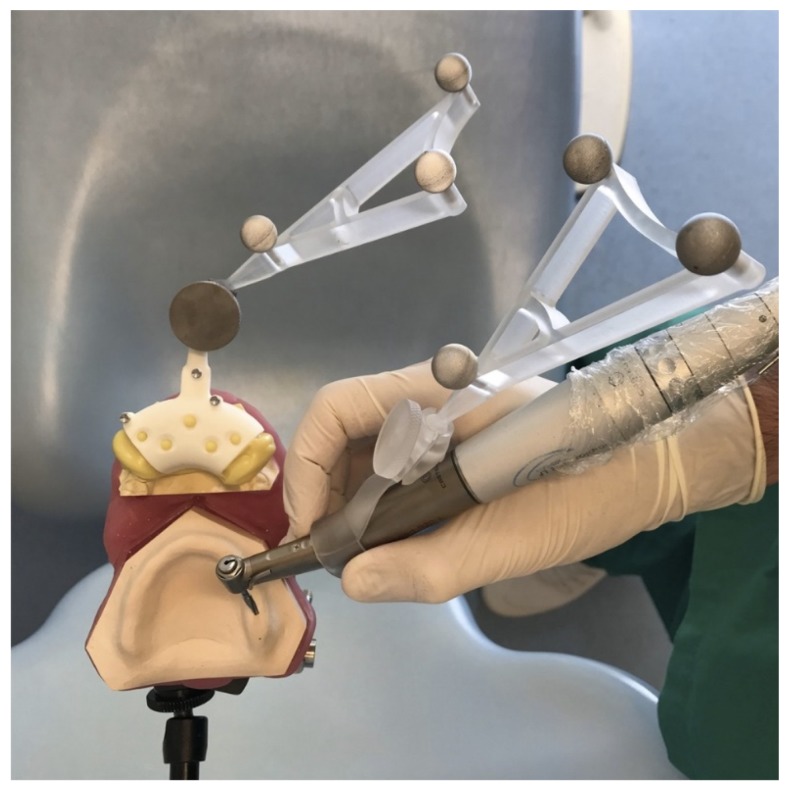
Drilling procedure. The reference tool is positioned on the model’s base and the tracking tools are placed on the reference tool and on the handpiece.

The sample size was calculated using G*Power 3.1 (Heinrich-Heine Universität, Düsseldorf, Germany), considering a power of 0.80, an alpha error of 5% and an f^2^ effect size of 0.08, based on previous studies [9,11]. The models were randomly assigned to four operators with different levels of experience in implantology and knowledge of dynamic navigation. The allocation ratio was balanced, so each operator performed 28 implant site preparations on four models. Operator number 1 (GP) was an oral surgeon with more than 10 years of experience in implant surgery (more than 2000 performed implant surgeries) and familiarity with the navigation system; operator number 2 (UC) was an oral surgeon with more than 10 years of experience in implant surgery (more than 2000 performed implant surgeries) and no familiarity in navigation surgery; operator number 3 (AZ) had performed less than 50 implant surgeries but had familiarity with the navigation system; operator number 4 (PFC) was a novice with no experience in implant surgery, nor in dynamic navigation. To replicate the patient’s position, the models were reproducibly fixed to the headboard of a dental chair on a customized acrylic support (Figure 2).

**Figure 3:**
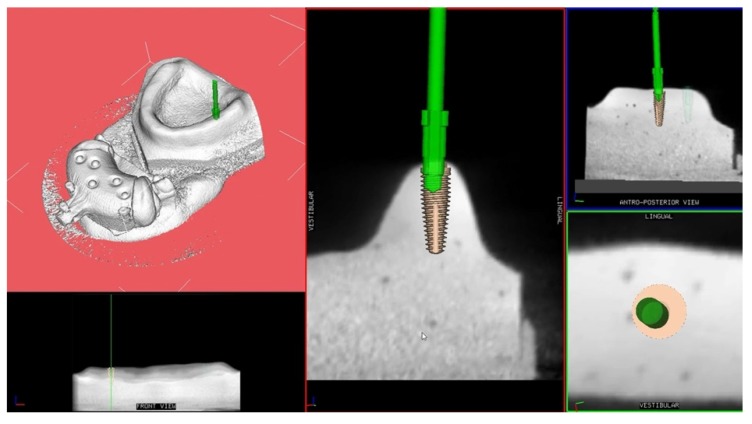
Screen display of the navigation system during implant site preparation.

The drilling procedure was performed with conical shaped drills (Southern Implants, Irene, South Africa) calibrated on a 4 × 11.5 mm implant using a dynamic navigation system (ImplaNav, BresMedical, Sydney, Australia). Thanks to the navigation system, the operator was able to follow the drill position in real-time on the system screen displaying the CBCT images of the model and the implant plan (Figure 3). 

**Figure 4:**
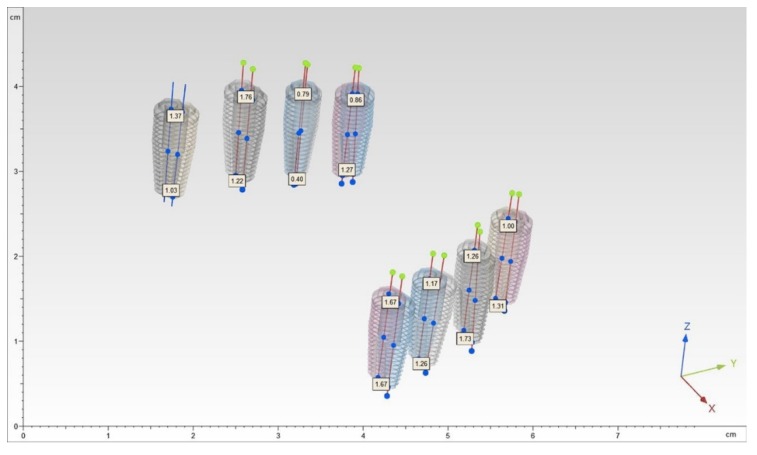
Superimposition of the preoperative and postoperative CBCTs and measurements of the deviations between the planned and the placed implants.

The time of each implant site preparation was measured. Then each model underwent a second CBCT scan. The DICOM data of the postoperative CBCT were imported to the software. From the digital library, dental implants (4 × 11.5 mm, conical, external hexagon) were chosen to optimally fit the drilled site. The implants were virtually positioned into every preparation site. Then the two CBCTs were superimposed and a blind outcome assessor quantified the accuracy, measuring the deviations between every planned implant and the virtually positioned one (Figure 4).

### 2.1. Outcome Assessment

**Figure 5:**
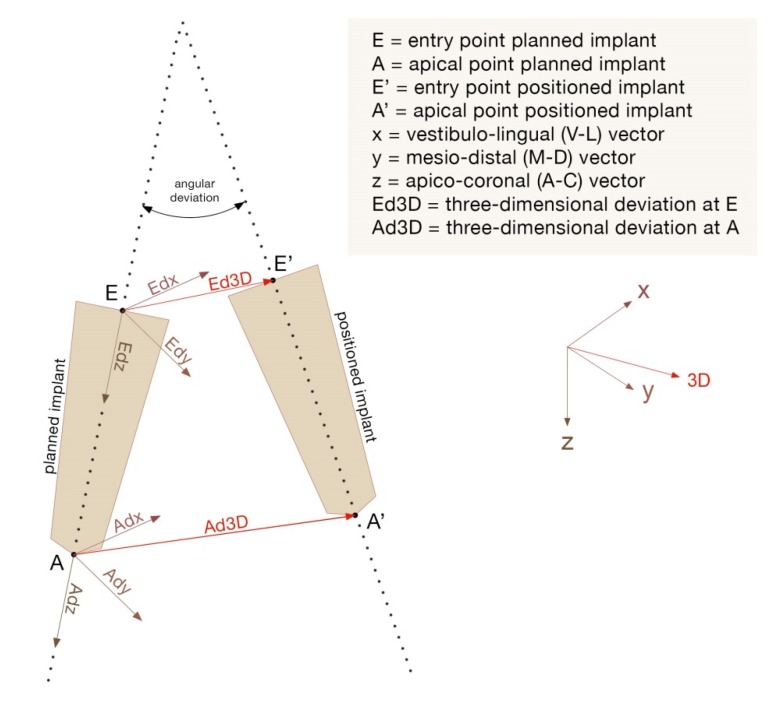
Representation of the 2D, 3D and angular deviations.

For each implant surface, two points were identified by the intersection between the symmetry axis and the implant surface: the apical point (A) and the entry point (E). The distances between the planned implants and the positioned ones for each pair of corresponding points were identified. They were decomposed into linear components identifying three euclidean vectors: x = bucco-lingual vector (V-L); y = mesio-distal vector (M-D); z = apico-coronal vector (A-C). A line was drawn for each implant by calculating the inertial axis. The angular deviation, expressed in degrees, was calculated by measuring the angle of discrepancy between two corresponding lines. The vectors for both coronal and apical points identified 2D deviations (Adx = apical V-L deviation; Ady = apical M-D deviation; Adz = apical depth deviation; Edx = coronal V-L deviation; Edy = coronal M-D deviation; Edz = coronal depth deviation). The three-dimensional accuracy was provided by 3D errors (3D E and 3D A). A blind outcome assessor measured these deviations quantifying the implant placement errors. The following dependent variables were considered: Adx, Ady, Ad, Edx, Edy, Edz, 3D E, 3D A and angular errors (Figure 5). Accordingly, coronal deviations, apical deviations, vertical deviations and angular deviations were identified. 

### 2.2. Statistical Analysis

A descriptive analysis of the accuracy values and drilling times was performed using means and standard deviations. After the verification of the assumptions of normality with a Shapiro–Wilk test and homoscedasticity with Levene’s test, a MANOVA was performed to test the hypothesis of equality between the four operators in terms of all the implant placement deviations. Then, a multiple comparison Bonferroni test was employed. A one-way ANOVA was carried out to test the differences between the operators in terms of drilling times. The level of significance for all the statistical tests was set at 5%. A blind researcher analyzed the data using Stata 15 (StataCorp LLC, Texas, TX, USA).

## 3. Results

### 3.1. Accuracy

A total of 112 measurements were analyzed. All the variables showed normal distributions, indicated by the *p*-values of the Shapiro–Wilk test of > 0.05. Non-significant values of the Levene’s test (*p* > 0.05) indicate equal variance between groups. Means and standard deviations of the 2D and 3D linear errors measured at the entry point and at the apical point and the angular errors are shown in Table 1. Considering both coronal and apical 3D deviations, no statistically significant differences were found between the four operators (*p* = 0.27; *p* = 0.06). The implant placement 3D errors at the entry point were 1.55 ± 1.08 mm for operator 1 (experienced and with good knowledge of the dynamic navigation system), 1.68 ± 0.69 mm for operator 2 (experienced and using the dynamic navigation system for the first time), 1.35 ± 0.67 mm for operator 3 (not experienced and with good knowledge of the dynamic navigation system) and 1.74 ± 0.64 mm for operator 4 (novice and without knowledge of the dynamic navigation system). The 3D errors measured at the apical point were 1.44 ± 0.95 mm for operator 1, 1.47 ± 0.68 mm for operator 2, 1.59 ± 0.74 mm for operator 3 and 1.92 ± 0.51 mm for operator 4. The overall 3D deviation measured was 1.58 ± 0.80 mm at the entry point (3D E) and 1.61 ± 0.75 mm at the apical point (3D A). Regarding the vectorial measures, no statistically significant differences were found between the operators in terms of the vertical component of both the deviations measured at the entry point and at the apical point (Adz, Edz). None of the three vectorial errors measured at the entry point (Edx, Edy, Edz) differed statistically. No statistically significant differences were found between operators 1, 2 or 3 in terms of Adx deviation, while operator 4 achieved a significantly higher value with respect to the other operators (*p* < 0.05). Regarding the Ady deviation, only operators 1 and 3 differed statistically (*p* = 0.001). Operator 4 had significantly higher values of angular deviation with respect to operators 1 (*p* = 0.000) and 2 (*p* = 0.002). 

### 3.2. Drilling Time

Drilling time measurements are shown in Table 2. Operator 1 had a statistically significantly lower drilling time with respect to operators 3 (*p* = 0.002) and 4 (*p* = 0.014). No significant differences were found when operator 4 was compared to operator 3 (*p* = 1.0), or when operator 3 was compared to operator 2 (*p* = 0.20). Operator 2 did not have a significantly different drilling time with respect to the other operators. 

## 4. Discussion

Few in vitro studies involving dynamic navigation systems aim to investigate the relevance of surgical experience in achieving an accurate implant placement [11,12]. 

The goal of the present study was to explore the association between operators’ experience and the definite outcomes: implant placement accuracy and drilling time. To do that, the implant placement errors of four operators with different levels of experience and knowledge of dynamic navigation were compared and the discrepancy in the implant site preparation time was evaluated. 

Considering all the involved clinicians, good accuracy values were found, comparable with those reported in other model-based studies [11,14,15,16]. The overall 3D deviation measured at the entry point (3D E) was 1.58 ± 0.80 mm and the overall 3D deviation measured at the apical point (3D A) was 1.61 ± 0.75 mm. 

Similarly, an in vitro study by Jorba-García et al. [11] reported an overall coronal 3D mean deviation of 1.29 ± 0.46 mm and an overall apical 3D mean deviation of 1.33 ± 0.50 mm. These authors assessed the implant placement accuracy of a skilled surgeon and a student, using a dynamic navigation system and a conventional free-hand technique. They stated that the use of a dynamic navigation system increased the capacity to place implants in a more accurate way compared to a free-hand technique, particularly if the operator is a novice. Nevertheless, they did not directly compare the accuracy values of the skilled operator and the inexperienced one. 

On the contrary, in the present study, the difference in terms of implant placement accuracy between clinicians with different surgical experience was tested. Even though some error measures were statistically lower for operator 1, these discrepancies appeared clinically irrelevant. Moreover, the 3D deviations did not statistically differ between all the groups, neither at the apical point nor at the coronal point, indicating that the lack of surgical experience and dynamic navigation knowledge did not substantially affect these accuracy values. 

All the measures of accuracy, including all the linear vectors of errors and the 3D deviations, are provided because the comparison of the results between studies is an arising issue in the literature concerning computer-guided implantology. In fact, a variety of measure types are presented and the lack of outcome uniformity could lead to a bad interpretation and to increased difficulty in obtaining pooled estimates.

The employed study design (in vitro) might limit the generalization of the results, namely the external validity. In fact, in a clinical environment, different variables could impair the performance of implant surgery and consequently the implant placement accuracy. Another limitation of this study is the lack of focus on other variables, like implant position (anterior/posterior), because of the sample size. 

On the other hand, good internal validity was provided by analyzing the pure effect of experience on implant placement accuracy without confounders that are harder to control in a clinical context. All the anatomical and operative variables were the same for all the clinicians and, moreover, the assessment of the outcomes was kept blind, preventing detection bias.

Regarding drilling time, two published model-based studies investigated the learning curve using dynamic navigation, but they lacked considerations on the influence of surgical experience [12,16]. 

In fact, Sun et al. [16] show that, as the experiment proceeds, the operating time is reduced and the level of accuracy reaches a plateau, after which improvements become less evident. However, the operator’s background is neither specified nor taken into account, precluding the evaluation of the surgical experience’s influence over the outcomes. 

Neither did Golob Deeb et al. [12] take this aspect into consideration, focusing only on students and documenting an increase in accuracy values and a reduction in operating time from the first to the last implant site preparation. 

In the present study, how level of surgical experience and knowledge of the navigation technique could affect drilling time using a dynamic navigation system was anlyzed. 

The mean drilling time of the skilled surgeon with insight into dynamic navigation was shorter. However, even though the differences between this surgeon and the inexpert ones were statistically significant, the discrepancies appeared negligible from a clinical point of view. Furthermore, the difference in drilling time between the two inexpert operators was not statistically significant, suggesting that a novice in implant surgery using a navigation system for the first time could also reach good results in terms of operating time, and still provide good accuracy values. Thus, knowledge of this guided technique did not seem to affect the speed of implant site preparation. In fact, the two skilled surgeons achieved similar results in terms of drilling time. 

The results of the present study suggest that dynamic navigation could be considered a reliable, easy-to-learn technique even in novices’ hands, allowing good results in terms of both accuracy and the speed of implant site preparation. Furthermore, when this technique is used by an expert surgeon, even if he has never tried it before, it seems to guarantee a good performance in terms of both accuracy and drilling time. In fact, the deviation measures and the drilling time did not differ from the values achieved by the skilled operator who is trained in navigation.

The findings and the hypothesis coming from this study should be considered a cue for future clinical research and must be validated by studies with a higher evidence level.

## 5. Conclusions

Dynamic navigation supports implant surgeons in achieving good results in terms of accuracy in implant site preparation. This issue seems to be independent from the operators’ skills in implantology and their knowledge of navigated surgery.

## Figures and Tables

**Table 1:** Implant placement deviations (mean ± standard deviation).

Operator	Coronal Deviation (mm)				Apical Deviation (mm)				Angular Deviation (°)
	Edx	Edy	Edz	3D E	Adx	Ady	Adz	3D A	
**1**	0.77 ± 0.60 ^a^	0.65 ± 0.39 ^a^	0.88 ± 1.12 ^a^	1.55 ± 1.08 ^a^	0.73 ± 0.49 ^a^	0.53 ± 0.37 ^a^	0.72 ± 0.91 ^a^	1.44 ± 0.95 ^a^	2.93 ± 1.50 ^a^
**2**	0.84 ± 0.53 ^a^	0.87 ± 0.67 ^a^	0.85 ± 0.61 ^a^	1.68 ± 0.69 ^a^	0.53 ± 0.43 ^a^	0.81 ± 0.61 ^ab^	0.84 ± 0.66 ^a^	1.47 ± 0.68 ^a^	3.54 ± 2.33 ^a^
**3**	0.54 ± 0.42 ^a^	0.91 ± 0.75 ^a^	0.51 ± 0.40 ^a^	1.35 ± 0.67 ^a^	0.58 ± 0.44 ^a^	1.17 ± 0.81 ^b^	0.48 ± 0.40 ^a^	1.59 ± 0.74 ^a^	4.51 ± 2.74 ^ab^
**4**	0.81 ± 0.52 ^a^	1.06 ± 0.71 ^a^	0.75 ± 0.57 ^a^	1.74 ± 0.64 ^a^	1.26 ± 0.64 ^b^	0.84 ± 0.54 ^ab^	0.77 ± 0.60 ^a^	1.92 ± 0.51 ^a^	5.90 ± 2.38 ^b^
**Tot**	0.74 ± 0.53	0.87 ± 0.65	0.75 ± 0.74	1.58 ± 0.80	0.78 ± 0.58	0.84 ± 0.64	0.70 ± 0.67	1.61 ± 0.75	4.24 ± 2.52

Edx: coronal V–L deviation; Edy: coronal M–D deviation; Edz: coronal depth deviation; 3D E: 3D coronal deviation; Adx: apical V–L deviation; Ady: apical M–D deviation; Adz: apical depth deviation; 3D A: 3D apical deviation. Letters a, b and ab indicate statistically significant difference or equality.

**Table 2:** Drilling time measurements.

Operator	N	Mean (s)	Standard Deviation	Standard Error	Mean Confidence Interval 95%		Min (s)	Max (s)
					Lower Limit	Upper Limit		
1	28	43.35 ^a^	14.75	2.78	37.63	49.07	24.00	83.69
2	28	51.50 ^ab^	19.35	3.65	44.00	59.01	28.18	96.14
3	28	62.47 ^b^	16.97	3.20	55.89	69.05	28.74	95.24
4	28	58.32 ^b^	24.05	4.54	49.88	68.53	32.15	125.00
Tot	112	54.13	20.24	1.91	50.34	57.92	24.00	125.00

N: number of implant site preparations; Mean (s): mean drilling time expressed in seconds; Min (s): minimum drilling time expressed in seconds; Max (s): maximum drilling time expressed in seconds. Letters a, b and ab indicate statistically significant difference or equality.
